# Promising High Monetary Rewards for Future Task Performance Increases Intermediate Task Performance

**DOI:** 10.1371/journal.pone.0042547

**Published:** 2012-08-08

**Authors:** Claire M. Zedelius, Harm Veling, Erik Bijleveld, Henk Aarts

**Affiliations:** Department of Social Psychology, Utrecht University, Utrecht, The Netherlands; Brain and Spine Institute (ICM), France

## Abstract

In everyday life contexts and work settings, monetary rewards are often contingent on future performance. Based on research showing that the anticipation of rewards causes improved task performance through enhanced task preparation, the present study tested the hypothesis that the promise of monetary rewards for future performance would not only increase future performance, but also performance on an unrewarded intermediate task. Participants performed an auditory Simon task in which they responded to two consecutive tones. While participants could earn high vs. low monetary rewards for fast responses to every second tone, their responses to the first tone were not rewarded. Moreover, we compared performance under conditions in which reward information could prompt strategic performance adjustments (i.e., when reward information was presented for a relatively long duration) to conditions preventing strategic performance adjustments (i.e., when reward information was presented very briefly). Results showed that high (vs. low) rewards sped up both rewarded and intermediate, unrewarded responses, and the effect was independent of the duration of reward presentation. Moreover, long presentation led to a speed-accuracy trade-off for both rewarded and unrewarded tones, whereas short presentation sped up responses to rewarded and unrewarded tones without this trade-off. These results suggest that high rewards for future performance boost intermediate performance due to enhanced task preparation, and they do so regardless whether people respond to rewards in a strategic or non-strategic manner.

## Introduction

The promise of rewards, such as money, for good performance is a powerful tool to get the best out of people, especially when the to-be-performed task is boring, repetitive, or otherwise not intrinsically interesting [1]–[4]. In line with this principle, rewards are widely used to entice people to increase their performance, that is, the speed or accuracy with which people execute a task. For instance, corporations offer monetary bonuses, schools offer awards, and sports tournaments offer prizes. Importantly, more often than not, such real-life rewards are not attainable right away, but are contingent on *future* performance. Nevertheless, previous research on the effects of rewards has focused exclusively on rewards that can be earned immediately. As a result, it is currently unclear how the promise of future rewards affects performance on reward-unrelated tasks that people encounter in the mean time. The present research aims to provide first insight into this topic by examining how rewards for future performance impact performance in terms of speed and accuracy on *intermediate tasks*–tasks that are carried out after the promise of reward has been made, but before the reward can actually be earned.

In the present study, we test the hypothesis that the promise of rewards for future performance increases performance not only on the task in which the reward can be earned, but also on an intermediate task. We further explore the hypothesis that this boost is not strategic, but occurs as a consequence of increased preparation for a rewarded task. The main aim of the present study is to enhance our understanding of the mechanisms via which rewards shape performance. Moreover, this study also offers interesting practical implications. In work settings, for example, the promise of rewards to be earned in the future may prove a useful and efficient tool to raise performance immediately.

### Rewards Increase Preparation before they Can be Earned

Previous studies suggest that rewards enhance performance by increasing effort and attention toward a task, which facilitate the execution of goal-directed actions [5]–[7]. Importantly, however, increased attention does not only serve successful performance by facilitating the execution of tasks, but also by facilitating preparation. Preparation entails the allocation of attention to the type of stimulus to follow and the type of action to be performed, and takes place even before a person has the necessary information to actually produce the appropriate action [8]–[12]. And indeed, studies have shown that rewards can improve performance by enhancing task preparation [13], [14]. Thus, the facilitative effect of rewards on performance materializes already *before* people engage in the execution of a rewarded task. This raises the intriguing possibility that the promise of reward for future tasks facilitates performance already when people carry out an intermediate task, even though this task is not instrumental for attaining the reward.

Based on other previous research, one could argue that this hypothesis is rather counterintuitive. That is, in research on the effects of performance-contingent rewards, it is generally assumed that effort is directed specifically toward rewarded tasks or task dimensions, and not toward unrewarded activities [1], [4], [15], [16]. In fact, research shows that people are generally reluctant to invest unnecessary effort [17]–[20], a tendency that might prevent the potential depletion of mental resources needed for important tasks [21]. From this perspective, people should be inclined *not* to increase performance on an intermediate task when presented with rewards that can be earned only through future performance. However, research has also shown that when people pursue a future reward, the reward information is automatically maintained in memory and remains highly accessible until the reward is obtained [22]–[27]. Consequently, we predict that rewards for future performance may immediately lead to preparation for optimal performance–even on tasks that are non-instrumental to the reward.

Some evidence indeed suggests that preparation for future performance can cause immediate performance improvement. In one study, it was found that the anticipation of a difficult (vs. easy) future task led to increased performance on an unrelated intermediate task [28]. However, it is unclear whether the immediate performance boost was caused by the mere expectation of having to perform a difficult task, or by the preparation to perform especially well on the future task (e.g., because rewards at stake). The present study addresses this issue directly by testing the effects of high and low rewards for future task performance on intermediate task performance.

### Strategic and Non-strategic Performance Adjustments

A further critical issue tested in the present study is whether the predicted effect of future task rewards on intermediate task performance is indeed a consequence of the mere preparation for the rewarded future task, and not the result of a deliberate strategy to improve future performance by intentionally raising immediate performance. It may be conceivable that people employ such a strategy to act on the implicit theory that exerting effort now may have an energizing function, and thereby improve performance in the future (e.g., see [29]). However, if people would deliberately improve immediate performance in response to high rewards as part of a strategy, this would be a different mechanism than we predict. According to our prediction, preparation for a highly rewarded future task can improve immediate performance even when people do not act strategically.

To test the potential role of strategic responses to future rewards, we compared performance under a condition in which reward information could lead to strategic performance adjustments to performance under a condition preventing strategic performance adjustments. To do so, we presented the reward information either for a relatively long duration, rendering this information clearly visible, or we presented the reward information too briefly to be consciously perceived. Previous work suggests that very brief presentation of reward information leads to so-called initial reward processing [30], which is rather rudimentary and very quick, and can unconsciously boost task preparation and performance. However, only full reward processing, which relies on prolonged presentation of reward information, allows for strategic performance adjustments. Indeed, a number of studies have shown that both very short (i.e., 17 ms) and relatively long (i.e., 300 ms) presentation of high (versus low) rewards boost performance on various cognitive and physical tasks [31]–[35]. However, only the longer presentation was shown to elicit strategic responses (for a review see [30]). For instance, in one study, it was found that only relatively long, but not brief reward presentation caused people to make deliberate speed-accuracy trade-offs for high compared to low rewards [32]). Thus, if our hypothesis is true that high rewards for future performance improve intermediate task performance in a non-strategic manner, we should observe a boost in performance on intermediate task performance even when rewards are presented too briefly to be consciously seen.

### The Present Research

To test the effects of future rewards on immediate performance, we used a reaction time task in which we rewarded fast reactions. Specifically, we used an auditory Simon task, in which participants were asked to quickly respond with a right versus left key to the pitch of a tone played through headphones to the right or left ear [36], [37]. We chose this task because it contains an irrelevant stimulus dimension (i.e., the side on which the tone is presented), which renders it demanding in terms of task preparation (e.g., [38], [39]), and previous work indicates that task performance under this condition is sensitive to monetary rewards [27]. The irrelevant stimulus dimension also creates congruent trials (i.e., trials on which the side to which the tone is presented matches the to-be-performed response) and incongruent trials (i.e., trials on which the side to which the tone is presented does not match the to-be-performed response). Previous work has found that rewards do not differently speed up responses to congruent and incongruent trials (e.g., [27], [40]), suggesting that (at least in these kinds of tasks) rewards improve task performance through greater task preparation rather than through online adjustments in conflict resolution. Accordingly, we did not expect rewards to differentially affect performance as a function of congruency. Finally, we imposed a demanding response time criterion that had to be met in order to obtain a reward. As explained below, this criterion was included to reveal performance differences between strategic and non-strategic reward processing.

On each trial, we first presented participants with a high (50 cents) or low value (1 cent) coin, followed by a series of two successive tones. The coin was presented between masks, and was shown either very briefly (17 ms) or for a relatively long duration (300 ms). Participants were instructed that rewards could be obtained for sufficiently fast accurate responses (according to a pre-specified response time criterion) to every second tone, while the speed and accuracy of responses to every first tone were irrelevant for obtaining the reward. Because high (vs. low) rewards increase task preparation [13], [14], and because even before the first tone was presented, participants already knew that they had to respond quickly to the second tone in order to get the reward, preparation for fast responses for high compared to low rewards could take place as soon as the reward was presented [8]–[11]. Therefore, we predicted that high (vs. low) rewards would lead to faster responses to both the first and the second tone in a series, even though first response was not instrumental for the reward.

Because we emphasized the importance of speed by imposing a strict response time criterion for obtaining rewards, we did not predict that high compared to low rewards would also improve the accuracy of rewarded and unrewarded responses. In fact, under the condition where participants were able to strategically adjust performance in reaction to the reward, we predicted the opposite. That is, strategic responding to high rewards should lead to a speed accuracy trade-off–a sacrifice of accuracy for greater speed [41], [42]. Importantly, and in line with our hypothesis that preparation for rewarded future performance affects immediate performance, we predicted that this strategy may already become apparent during intermediate task performance. Therefore, when participants responded strategically to rewards, a speed accuracy trade-off in response to high rewards should occur not only for the second, rewarded tone, but also for responses to the first, unrewarded tone. Because speed accuracy trade-offs induced by high rewards only occur when rewards are presented relatively long and can be fully processed [32], we did not expect this speed accuracy trade-off in response to high rewards when the reward information was presented briefly.

To sum up, our first hypothesis was that high vs. low rewards contingent on fast responses to the second in a series of two tones would speed up rewarded responses to this second tone as well as unrewarded responses to the first tone. Furthermore, this speed-up should occur regardless whether reward information was presented for a relatively long duration, allowing for strategic responses and speed accuracy trade-offs, or very briefly, limiting strategic responding.

## Methods

### Ethics Statement

The experiment was conducted with healthy human participants, and did not utilize any invasive techniques, substance administration or psychological manipulations. Therefore, compliant with Dutch law, this study only required and received approval from the local faculty board at Utrecht University. The study was conducted, and written informed consent of each participant was obtained in compliance with the principles contained in the Declaration of Helsinki.

### Participants and Design

Participants were 91 university students (64 women) with a mean age of 20.49 (*SD* = 2.39). The design was a within-subjects design with the factors reward (high vs. low), exposure (long vs. short), and congruency of the ear–response key combinations (congruent vs. incongruent). Reward and exposure varied randomly over trials (i.e., a trial involved presentation of two tones), and the factor congruency varied randomly for both the first and the second tone within a trial. Dependent measures were reaction times (RTs) and accuracy of responses to the first tone and to the second tone.

### Materials and Methods

The experimental task was programmed and run using the software package e-Prime 1.2 (Psychology Software Tools Inc., Pittsburgh, Pennsylvania, USA; see [43]). The timing of stimulus presentation was synchronized with the vertical retraces of a 60-Hz monitor, resulting in a vertical refresh rate of 16.67 ms.

### Procedure

Participants performed an auditory Simon task [36], in which they were presented repeatedly with series of high and low pitch tones. For each tone, participants were asked to indicate with a right or left key on the keyboard whether the tone was of high or low pitch (the assigned keys were counterbalanced between participants). Participants were instructed that they would repeatedly be presented with a series of two consecutive tones, and that they could obtain monetary rewards for very fast correct responses on each second tone in a series. They were further told that responses to each first tone in a series were irrelevant for obtaining rewards. To emphasize the need for fast responding, rewards could only be earned if responses to the second tone on a trial did not exceed a pre-specified response time (RT) criterion of 350 ms. The time for this RT criterion was set below the average RT found in previous studies employing this task (e.g., [44], [45]), and was based on pilot data showing that this criterion yielded about 75% sufficiently fast responses on rewarded trials.

To familiarize participants with the task setup and the RT criterion, participants were given a practice round. The setup of the practice round was identical to the experimental task (see below). All participants completed at least one practice block consisting of 10 trials. If participants did not meet the RT criterion on any trial in this practice block, they performed additional practice blocks of each 5 trials until they met the RT criterion on each trial.

**Figure 1:**
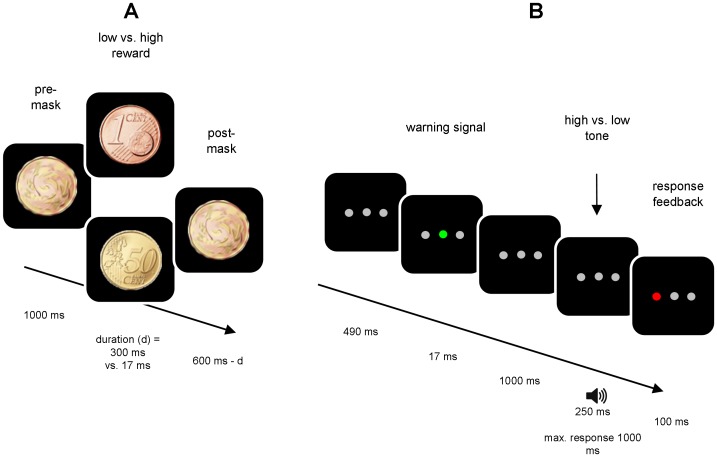
Schematic overview of the experimental task. Each trial started with the presentation of a reward, sandwiched between a pre- and a post-mask (A), followed by two repetitions of the auditory Simon task (2×B). Between the two repetitions, a blank screen was presented for the duration of 1500 ms minus the time of the response to the first tone (if the response time exceeded 1500 ms, the task would continue automatically).

The experimental task consisted of 32 trials. Each trial started with an empty screen shown for 1000 ms, followed by the presentation of a reward in the form of a coin of either 1 cent or 50 cents, which was presented for either 300 ms (supraliminal condition) or 17 ms (subliminal condition). The coins were preceded by a pre-mask presented for 1000 ms and followed by a post-mask for 600 ms minus the duration of the presentation time of the coin. The coins and masking stimuli are depicted in Figure 1.

To verify that the coins in the short presentation condition were presented too quickly to be consciously visible, limiting the opportunity for strategic responding (see [46]), a subliminality test was performed: An independent sample of 31 participants were presented with 1 cent and 50 cents coins shown for 17 ms with a pre mask and a post mask, following the same procedure as in the experiment. Participants were asked to indicate for each coin whether they had seen a 1 cent or 50 cents coin. A t-test confirmed that identification of the coins was at chance (*M* = 0.52, *SD* = 0.12), *t* (30) = 0.79, *p* = .44.

In the experimental task, after the presentation of the coin, a black screen with three horizontally positioned gray circles was presented for 490 ms. Next, a green flash appearing in the middle circle served as a warning signal for the first upcoming tone. 1000 ms after this warning signal, the first high (500 Hz) or low pitch (200 Hz) tone was played for 250 ms. The tone was followed by a response window of 1000 ms, in which participants could respond with a key press. If the RT exceeded 1000 ms, the task continued automatically. Visual response feedback was given via a red flash for 100 ms in the (left or right) circle corresponding to the side of the response. Next, a blank screen was presented for the duration of 1500 ms minus the RT for the first tone. The purpose of the flexible timing of this interval was to ensure that the second tone would occur at a fixed time after the reward information, regardless how fast participants responded to the first tone. After this interval, a second warning signal and a second tone were presented, following the same procedure as before, and again followed by an identical response window and response feedback. Finally, after the second response, performance feedback was shown for 1300 ms, informing the participant about the amount of reward (0, 1, or 50 cents) received for their response to the second tone. This marked the end of a trial. For an overview of the task procedure, see Figure 1.

After the task, participants were asked to answer two questions concerning the experimental task. The first question served as an instruction check. Specifically, participants were asked whether they had been aware that the rewards presented during the task could be earned for fast and correct responses to the *second* but not the *first* tone of a trial. Answers were given on a scale from 1 (“no not at all”) to 7 (“yes, very much”). Second, participants were asked whether, for each tone they had had heard, it was clear whether it had been the first or the second in a series of tones. Answers were given on a scale from 1 (“no, never”) to 7 (“yes, every time”). After answering these questions, participants were thanked for their participation and dismissed.

## Results

To test our experimental hypotheses, we first present the results for the second, rewarded tone and the first, unrewarded tone separately. Then, we present an analysis examining the effects on both tones in one design in order to compare them. Finally, we present analyses involving the post-experimental questions in order to rule out potential alternative explanations.

### Performance in Response to the Second, Rewarded Tone

#### Response times

RTs from incorrect responses (*M* = 12.29%, *SD* = 13.05) were removed from the response time analyses. Responses that were correct but exceeded the RT criterion of 350 ms, and were thus not rewarded (M = 27.44%, *SD* = 24.66%), were included in the analysis. (See below for additional analyses without RTs exceeding 350 ms). An initial repeated-measures ANOVA with the factors reward, exposure and congruency yielded a congruency effect, entailing that responses to congruent ear-response key combinations (*M* = 296.87, *SD* = 54.49) were faster than those to incongruent combinations (*M* = 277.97, *SD* = 59.18), *F* (1, 74) = 110.69, *p*<.001, *η_p_^2^* = 0.60. Congruency did not interact with any of the other factors (all *F*s<1.25). Because the removal of incorrect responses for some participants led to empty cells in the design, and hence reduced the power of the analysis, in the following analyses data were collapsed over the factor congruency.

**Figure 2:**
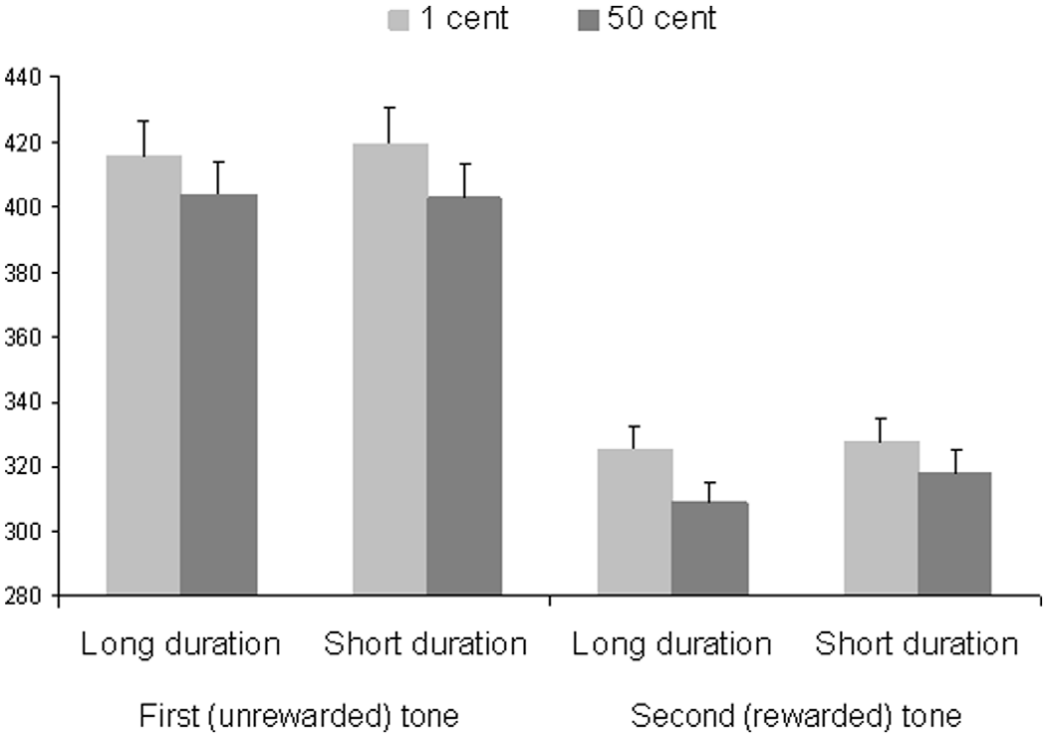
Mean reaction times of responses to the first (unrewarded) and second (rewarded) tone in a series as a function of reward value and reward presentation duration. Error bars represent standard errors.

A repeated-measures ANOVA with the factors reward and exposure yielded the predicted main effect of reward, *F* (1, 90) = 12.59, *p* = .001, *η_p_^2^* = 0.12, showing that, when fast responses were rewarded, high (*M* = 313.33, *SD* = 55.31) compared to low (*M* = 326.55, *SD* = 62.34) rewards led to faster responses (see Figure 2). There was no main effect of exposure, *F* (1, 90) = 2.49, *n.s.*, and no interaction of reward × exposure, *F* (1, 90) = 1.39, *n.s.,* indicating that response speed did not differ for relatively long and briefly presented reward information.

Because performance was only rewarded when RTs were correct and below 350 ms, we repeated the above reported analyses after excluding incorrect responses as well as responses exceeding 350 ms. (Note that exclusion of these RTs led to empty cells for a number of participants, and thus reduced the number of participants in the analyses.) The results were similar to those reported above. Specifically, a repeated-measures ANOVA with the factors reward and exposure yielded a main effect of reward, *F* (1, 81) = 8.37, *p* = .005, *η_p_^2^* = 0.09 (high rewards: *M* = 300.81, *SD* = 39.00; low rewards: *M* = 311.51, *SD* = 39.24) but no main effect of exposure, *F* (1, 81)  = 1.21, *n.s.*, and no interaction of reward × exposure, *F* (1, 81) = 0.38, *n.s.*

#### Accuracy

Accuracy scores were first subjected to a repeated-measures ANOVA with the factors reward, exposure and congruency. This yielded a main effect of congruency, *F* (1, 90) = 49.60, *p* = .001, *η_p_^2^* = 0.36, indicating that participants made less incorrect responses for congruent (*M* = 0.90, *SD* = 1.15) than incongruent ear-response key combinations (*M* = 3.03, *SD* = 2.53). Congruency did not interact significantly with any of the other factors (all *F*s<1.41), so we again collapsed over congruency in the following analyses.

**Figure 3:**
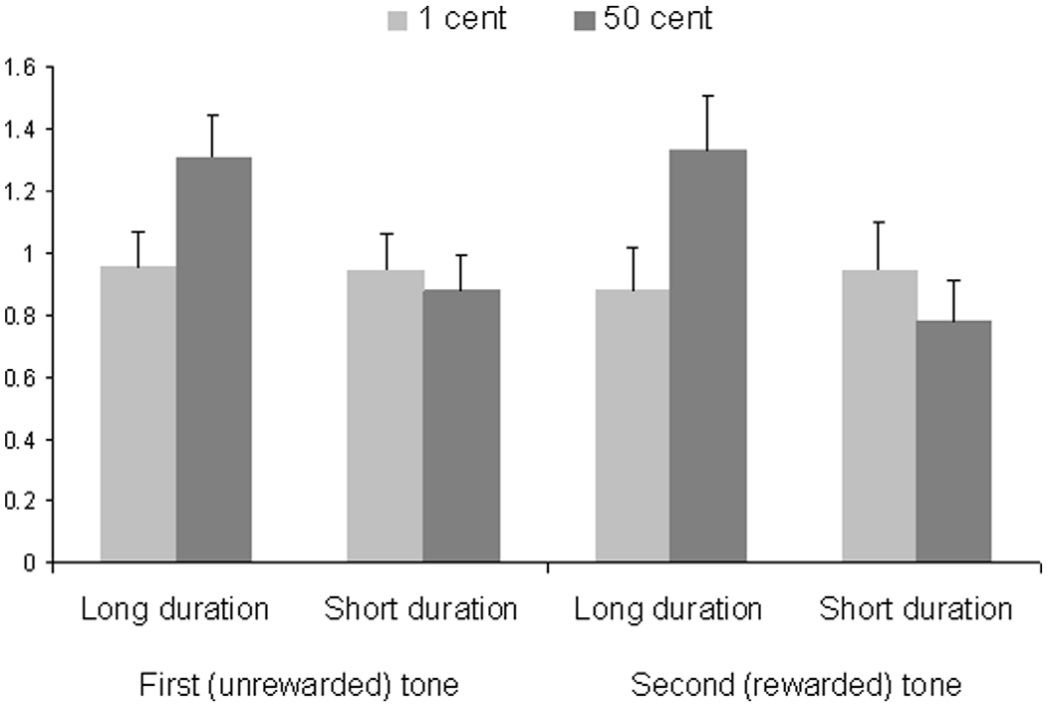
Mean number of incorrect responses to the first (unrewarded) and second (rewarded) tone in a series as a function of reward value and reward presentation duration. Error bars represent standard errors.

A repeated-measures ANOVA with the factors reward and exposure yielded no main effect of reward, *F* (1, 90)  = 1.34, *p* = .25. There was, however, a main effect of exposure, *F* (1, 90) = 4.57, *p* = .04, *η_p_^2^* = 0.05, indicating that participants made more incorrect responses on trials with long (*M* = 2.21, *SD* = 2.47) compared to short reward presentation (*M* = 1.73, *SD* = 2.22). This effect was qualified by an interaction of exposure × reward, *F* (1, 90) = 6.61, *p* = .01, *η_p_^2^* = 0.07 (See Figure 3). Simple effects analyses showed that when rewards were presented for a relatively long duration, high rewards (*M = *1.33, *SD* = 1.69) led to more incorrect responses than low rewards (*M = *0.88, *SD* = 1.32), *F* (1, 90) = 6.10, *p* = .02, *η_p_^2^* = 0.06. For briefly presented rewards, this effect was absent, *F* (1, 90) = 1.05, *n.s.* (high rewards: *M = *0.78, *SD* = 1.24; low rewards: *M = *0.95, *SD = *1.46).

### Performance in Response to the First, Unrewarded Tone

#### Response times

To test our hypothesis that rewards for quick responses to each second tone in a series would increase the speed of responses to *preceding* tones, we subjected RTs of each first, unrewarded tone to a repeated-measures ANOVA with the factors reward, exposure, and congruency. RTs from incorrect trials (*M* = 12.77%, *SD* = 10.95%) were removed from the analysis. The analysis yielded the typical congruency effect (RTs for congruent combinations: *M* = 381.22, *SD* = 94.80; RTs for incongruent combinations: *M* = 440.74, *SD* = 87.76), *F* (1, 84) = 119.63, *p*<.001, *η_p_^2^* = 0.59). Again, congruency did not interact with any of the other factors (all *Fs*<1.34). Thus, we again collapsed over congruency in the following analyses.

A repeated-measures ANOVA with the factors reward and exposure yielded the predicted main effect of reward, *F* (1, 90) = 8.84, *p* = .004, *η_p_^2^* = 0.09, indicating that even when performance was not rewarded, high (*M* = 403.13, *SD* = 93.04) compared to low rewards (*M* = 417.59, *SD* = 94.81) for fast responses to later tones sped up intermediate responses (see Figure 2). Again, there was no interaction of reward × exposure, *F* (1, 90) = 0.21, *n.s.*

#### Accuracy

As for the rewarded responses, a repeated-measures ANOVA with the factors reward, exposure and congruency on accuracy scores showed a main effect of congruency, *F* (1, 90) = 8.05, *p* = .006, *η_p_^2^* = 0.08, indicating that participants again made less mistakes for congruent (*M* = 1.65, *SD* = 1.61) than incongruent ear-response key combinations (*M* = 2.44, *SD* = 2.66). Congruency did not interact with any of the other factors, (all *F*s<3.10), so we again collapsed over this factor in further analyses.

A repeated-measures ANOVA with the factors reward and exposure revealed a main effect of exposure, *F* (1, 90) = 4.57, *p* = .04, *η_p_^2^* = 0.05, indicating that participants made more incorrect responses when rewards were presented relatively long (*M* = 2.26, *SD* = 2.07) compared to when they were presented briefly (*M* = 1.82, *SD* = 1.83). Moreover, we again found an interaction of exposure × reward, *F* (1, 90) = 6.61, *p* = .01, *η_p_^2^* = 0.07 (see Figure 3), which showed that that when rewards were presented relatively long, high rewards (*M = *1.31, *SD* = 1.28) led to more incorrect responses than low rewards (*M = *0.96, *SD* = 1.07), *F* (1, 90) = 8.67, *p* = .004, *η_p_^2^* = 0.09. This effect was again absent for briefly presented rewards, *F* (1, 90) = 0.23, *n.s.* (high rewards: *M = *0.88, *SD* = 1.13; low rewards: *M = *0.95, *SD = *1.11).

### Performance in Response to the First and Second Tone

#### Response times

To directly compare the effects of rewards on performance on the first, unrewarded, and the second, rewarded tone, we performed an additional repeated-measures ANOVA including the factors reward, exposure, and tone (first vs. second) on RTs of correct responses the first and second tone. This yielded a main effect of tone indicating that RTs were generally faster on the second, rewarded tone (*M = *319.94, *SD* = 56.19) compared to the first, unrewarded tone (*M = *410.36, *SD* = 91.02), *F* (1, 90) = 182.00, *p*<.001, *η_p_^2^* = 0.67 (see Figure 2). Moreover, as for the separate analyses for the two tones, there was a main effect of reward, *F* (1, 90) = 18.00, *p*<.001, *η_p_^2^* = 0.17, and no interaction of reward × exposure, *F* (1, 90) = 0.03, *n.s.* Follow-up analyses further confirmed that the main effect of reward was significant regardless whether the reward was presented relatively long, *F* (1, 90) = 9.87, *p* = .002, *η_p_^2^* = 0.10, or briefly *F* (1, 90) = 7.04, *p* = .009, *η_p_^2^* = 0.07. Thus, even though participants responded faster to rewarded than unrewarded tones, reactions to the two tones were influenced in the same way by the long and briefly presented rewards.

#### Accuracy

The same analysis was performed on accuracy scores for both tones. This yielded no main effect of trial, *F* (1, 90) = 0.45, *n.s.,* but, as was found for the separate analyses for each tone, there was a main effect of exposure, *F* (1, 90) = 5.89, *p* = .02, *η_p_^2^* = 0.06, and an interaction of exposure × reward, *F* (1, 90) = 7.69, *p* = .007, *η_p_^2^* = 0.08. This interaction was not qualified by a three-way interaction of exposure × reward × trial, *F* (1, 90) = 1.26, *n.s*. This shows that the differential effects of long versus briefly presented rewards reported above did not differ for reactions to rewarded compared to and unrewarded tones.

### Post-experiment Questions

To examine whether the effects reported above might have been caused by possible confusion about the task instructions, we first explored participants’ answer to the question whether they were aware of the instruction that rewards were contingent on responses to the second but not the first tone. The average score on this question was 5.73 (*SD* = 1.73; 7-point scale). Although this score is quite high, not all participants provided the maximum score. This could mean that for some participants the instructions were not completely clear. However, it is also possible that posing the question evoked confusion or the suspicion that the first tone had been relevant for the reward after all, causing participants who had clearly understood the instructions to call their understanding into question. To make sure that the results were not caused by some participants’ insufficient understanding of the instructions, we repeated the above reported analyses, now including participants’ answer to this instruction question as a factor (“instruction”) using a median split. This allowed us to compare the above effects in a group of participants scoring below 7, for whom we cannot be absolutely certain that they were sufficiently aware of the instructions (mean score  = 4.53; *SD* = 1.68; N = 47), to those in a group of participants who scored 7, indicating that they were clearly aware of the instructions (N = 44). Results showed that the factor instruction did not significantly interact with any of the effects of long and briefly presented rewards on response speed and accuracy for responses to both tones (e.g., reward by instruction interaction on reaction times, *F* = 0.17).

Next, we analyzed participants’ answers to the question whether it was clear that a tone was the first or second in a trial. The average score was 6.35 (*SD* = 1.06; 7-point scale). This high score indicates that participants were generally not confused about which of the tones was the rewarded one. To provide more evidence for this postulation, we again repeated the above reported analyses on the effects of long and briefly presented rewards on performance on the first and second tone, now including participants’ answer to this tone-identification question as a factor using a median split. Again, this yielded one group scoring below 7 (mean score  = 5.36; *SD* = 1.10; N = 36), and one group scoring 7 (N = 55). The results showed that tone identification did not interact with any of the effects reported above (e.g., reward by tone-identification interaction on reaction times, *F* = 0.24).

## Discussion

Based on evidence that rewards enhance performance by increasing task preparation [13], [14], and that preparation starts before people actually engage in the execution of a task [8]–[11], the present study tested the novel hypothesis that rewards contingent on future performance lead to immediate performance enhancement on an intermediate task. We tested this hypothesis by offering relatively high and low value monetary rewards for fast responses to each second tone presented in a series of two tones. Crucially, we found that higher rewards sped up not only responses to the second tone, but also responses to the first tone in the series–despite the fact that these earlier responses were unrelated to obtaining the rewards. To our knowledge, this is the first study showing that rewards for future task performance increase performance immediately.

Furthermore, the present data show that the effects of future rewards on immediate performance occurred both when reward information was presented long enough to enable strategic performance adjustments, and when reward information was presented too quickly to be consciously perceived, thus limiting strategic performance adjustments. Importantly, a comparison of the accuracy data for long and short reward presentation durations revealed that participants indeed responded more strategically to rewards when these were presented for a longer duration. This strategic responding became apparent in a speed accuracy trade-off in response to high rewards. This finding conceptually replicates previous work, which also showed speed accuracy trade-offs under conditions of long but not short reward presentation [32]. What is new and particularly interesting about this finding is that the speed accuracy trade-off for long-presented high rewards occurred for both rewarded and unrewarded responses. This suggests that when people prepare a strategy for highly rewarded future performance, this strategy already becomes apparent during intermediate task performance.

As a potential alternative explanation for these effects, one may propose that participants in the current experiment responded in the same way to rewarded and unrewarded tones because they were not aware which tones were rewarded. We can rule out this alternative explanation on several grounds. First, an analysis of the full design showed that, while rewards had similar effects on rewarded and unrewarded task performance, overall responses were much slower for unrewarded than rewarded tones. This suggests that people did not try to perform particularly well on the unrewarded intermediate task. However, the overall slower unrewarded responses may in some way be attributable to the task setup. After all, unrewarded responses always preceded rewarded responses. And indeed, there is evidence from one study, also using an interference task, showing that when participants are presented with series of two consecutive stimuli intermitted by irrelevant neutral information, responses are faster for the second than on the first stimulus [47]. Thus, the slower responses to unrewarded tones in the present study alone are not sufficient to rule out the alternative explanation that participants were confused about which responses were rewarded. However, the data from our post-experiment questions indicate, first of all, that participants were well aware of which responses were rewarded and which ones were not. Moreover, analyses including participants’ answers to questions regarding the reward contingencies showed that differences in participants’ awareness of the reward-contingencies cannot account for the pattern of results observed in this experiment.

It is interesting to note that the performance-boosting effects of future rewards on immediate performance in this study did not differ as a function of congruency. The observation that rewards do not reduce congruency effects on response times has been found before (e.g., [27], [40]). This result is consistent with the notion that in response time tasks such as the one employed here, rewards affect performance via task preparation rather than via online-adjustments once response-relevant stimuli are encountered [27]. Another explanation for why rewards did not reduce the congruency effect in the current study may be the application of a strict and specific reward criterion based on response speed, which led to the specific improvement of response speed but not of other control processes. This argument is in line with research showing that when rewards are provided for a specific task dimension, only performance on that dimension is improved [1], [4], [15], [16].

The finding that rewards for future performance non-strategically enhanced immediate performance on an intermediate task has interesting and important implications for the ongoing discussion about the effectiveness of different incentive schemes [48]. Tying monetary incentives to specific tasks has powerful effects on performance. However, researchers warn that such incentive schemes, when applied in a work context, can harm performance in the long run, because they lead to underperformance on unrewarded tasks (e.g., [49], [50]). The present study provides a more nuanced view on this idea. While our data confirm previous studies showing that rewards improve performance selectively for rewarded task dimensions [1], [4], [15], [16], rewards appear to improve performance much less selectively when reward contingencies refer to timing – *when* performance is rewarded. Thus, promising rewards for performance on a specific task at a later time may prove an effective and efficient tool to raise immediate performance along the way.

An interesting question for follow-up research concerns potential limitations to the effects of future performance rewards on intermediate performance. For instance, it may be that these effects depend on the similarity between the intermediate task and the rewarded task. In the present study, the intermediate task was identical to the rewarded task. This setup resembles everyday life and work contexts where people are confronted with repetitive work. However, it is possible that rewards for future performance do not affect performance on an intermediate task in the same way when this task requires very different kinds of responses. We argue that the effect of future rewards on immediate performance is the result of preparation for the rewarded task. Preparation for one type of action likely does not facilitate *any* kind of action in an intermediate task. However, the boundary conditions of this preparation effect are difficult to assess. This is because even when tasks differ considerably, there is usually some degree of overlap in the processes required for good task performance. For instance, even when two tasks involve very different motor responses to different stimuli under different instructions, preparation for good performance on one task does not only include preparing the respective motor responses, but also processes such as the recruitment of executive control, effort, and concentration; processes that probably benefit both tasks. Thus, more research is needed to delineate the conditions under which future rewards affect intermediate task performance.

To conclude, as with any novel finding, the present study raises many new questions. Nonetheless, the present study is the first to show that the promise of future rewards boosts performance immediately, even when people know that immediate performance is not rewarded. More broadly, the present study suggests that the time course of reward effects on performance may prove a fruitful area for further investigation, and that reward effects in general may be more ubiquitous than was previously thought.
